# High-Resolution 3D *in vivo* Brain Diffusion Tensor Imaging at Ultrahigh Fields: Following Maturation on Juvenile and Adult Mice

**DOI:** 10.3389/fnins.2020.590900

**Published:** 2020-11-20

**Authors:** Maxime Yon, Qingjia Bao, Odélia Jacqueline Chitrit, Rafael Neto Henriques, Noam Shemesh, Lucio Frydman

**Affiliations:** ^1^Department of Chemical and Biological Physics, Weizmann Institute, Rehovot, Israel; ^2^Champalimaud Research, Champalimaud Centre for the Unknown, Lisbon, Portugal

**Keywords:** diffusion tensor imaging, brain tractography, mouse cerebellum, mouse olfactory bulb development, high field DTI

## Abstract

Diffusion tensor imaging (DTI) is a well-established technique for mapping brain microstructure and white matter tracts *in vivo*. High resolution DTI, however, is usually associated with low intrinsic sensitivity and therefore long acquisition times. By increasing sensitivity, high magnetic fields can alleviate these demands, yet high fields are also typically associated with significant susceptibility-induced image distortions. This study explores the potential arising from employing new pulse sequences and emerging hardware at ultrahigh fields, to overcome these limitations. To this end, a 15.2 T MRI instrument equipped with a cryocooled surface transceiver coil was employed, and DTI experiments were compared between SPatiotemporal ENcoding (SPEN), a technique that tolerates well susceptibility-induced image distortions, and double-sampled Spin-Echo Echo-Planar Imaging (SE-EPI) methods. Following optimization, SE-EPI afforded whole brain DTI maps at 135 μm isotropic resolution that possessed higher signal-to-noise ratios (SNRs) than SPEN counterparts. SPEN, however, was a better alternative to SE-EPI when focusing on challenging regions of the mouse brain –including the olfactory bulb and the cerebellum. In these instances, the higher robustness of fully refocused SPEN acquisitions coupled to its built-in zooming abilities, provided *in vivo* DTI maps with 75 μm nominal isotropic spatial resolution. These DTI maps, and in particular the mean diffusion direction (MDD) details, exhibited variations that matched very well the anatomical features known from histological brain Atlases. Using these capabilities, the development of the olfactory bulb (OB) in live mice was followed from week 1 post-partum, until adulthood. The diffusivity of this organ showed a systematic decrease in its overall isotropic value and increase in its fractional anisotropy with age; this maturation was observed for all regions used in the OB's segmentation but was most evident for the lobules' centers, in particular for the granular cell layer. The complexity of the OB neuronal connections also increased during maturation, as evidenced by the growth in directionalities arising in the mean diffusivity direction maps.

## Highlights

- High-fields and cryogenic coils enable mouse brain DTI with better than 100 μm isotropic resolution.
- SE-EPI yields DTI distortions in cerebellar and olfactory bulb regions, that are alleviated by SPEN acquisitions.
- The ensuing structures show excellent agreement with adult mouse brain atlas data.
- High-field SPEN DTI reveals the progression of mice's olfactory bulb microstructure from newborns to adults.

## Introduction

Diffusion Tensor Imaging (DTI) (Basser et al., 1994) provides important morphological information about microstructure in healthy tissues, as well as biomarkers for pathological conditions. DTI is typically based on the characterization of water's three-dimensional diffusivity, as described by a 3 × 3 tensor whose eigenvalues and eigenvectors reflect restricted molecular motions within microscopic environments. When applied to animal or human brains, these restrictions are dominated by the myelin sheaths associated to the axon fibers of white matter; DTI is therefore used to study white mater diseases (Pierpaoli et al., 1996; Moseley, 2002; Johansen-Berg and Behrens, 2009; Le Bihan and Johansen-Berg, 2013), and to track developing and established white matter pathways in both human and animal brains (Conturo et al., 1999; Mori et al., 1999; Basser et al., 2000; Behrens et al., 2007; Nucifora et al., 2007; Catani and Thiebaut de Schotten, 2008). To map the full tensor, diffusion weighted measurements need to be acquired along several directions; in some cases, when the diffusion behavior deviates from Gaussian, multiple diffusion weightings per direction are also desirable (Jensen et al., 2005). Furthermore, since diffusivity along each of these directions is measured via significant signal attenuations, the experiment has intrinsic sensitivity losses; all of this may call for some form of signal averaging. Signal-to-Noise Ratio (SNR) will be further compromised if seeking this information with a high spatial definition, involving concomitantly smaller number of emitting spins per voxel. The acquisition of DTI datasets is also challenged by deleterious effects of physiological motion (Mori and Zhang, 2006), which, in conjunction with the bipolar diffusion-weighting gradients, often lead to distortions that are incompatible with many common imaging sequences.

As a result of all this, the highest resolution DTI studies have usually been achieved *ex vivo* (Aggarwal et al., 2010; Roebroeck et al., 2018), where motion artifacts become irrelevant and extensive signal averaging can overcome sensitivity penalties. Simple spin-echo (SE) sequences possessing good tolerance to field inhomogeneities, have been commonly used in these tests (Roebroeck et al., 2018). *Ex vivo* studies, however, cannot substitute diagnostic or longitudinal *in vivo* studies (Sidaros et al., 2008); even research-oriented biological measurements can be affected by changes in water's diffusivity upon death (Widjaja et al., 2009), and/or by tissue fixation (Shepherd et al., 2009). *In vivo* DTI bypasses most common motion artifacts by using spin-echoed Echo Planar Imaging (SE-EPI) sequences, possessing high SNR per unit time and good robustness against motion when executed in their single-shot 2D versions (Mukherjee et al., 2008). Single-shot sequences based on Carr-Purcell-Meilboom-Gill (CPMG) schemes such as Fast/Turbo-Spin-Echo (FSE/TSE) or Rapid-Acquisition-with-Relaxation-Enhancement (RARE) are also sometimes used (Sarlls and Pierpaoli, 2008) –even though multiple echoes coupled to strong diffusion-weighting gradients may induce phase shifts that complicate these acquisitions (Mori and Van Zijl, 1998). Regardless of which of these approaches is used, spatial resolution remains an important limitation in DTI, including in murine models where the fields-of-view (FOVs) and morphological features are small. SE-EPI can partially overcome this drawback by relying on segmented or interleaved acquisitions (Butts et al., 1994; Robson et al., 1997; Heidemann et al., 2010; Alomair et al., 2015). An alternative combining attractive features of CPMG-based acquisitions with those of EPI is the GRASE (Gradient—and Spin-Echo) sequence (Oshio and Feinberg, 1991; Aggarwal et al., 2010); to our knowledge this has led to the highest *in vivo* DTI spatial resolutions obtained so far: 100 μm isotropic for a murine brain (Wu et al., 2013), and 80 μm isotropic for the cerebellum (Wu et al., 2014).

Two established strategies for improving SNR and thereby spatial resolution, include operating at the highest possible fields and employing cryogenically cooled probes. Higher fields should improve sensitivity ca. quadratically with B_0_ via Boltzmann and Faraday effects; while evidence for a supra-linear enhancement has indeed been found (Pohmann et al., 2016), increased fields will also entail shorter T_2_s and larger susceptibility distortions that can significantly detract from these enhancements—particularly when considering diffusion-weighted measurements, where the diffusion-weighting module puts a limit to how short echo times can be. The advantages of cryogenic coils for ultrahigh-field ^1^H experiments where noise is increasingly dominated by the sample's physiology and ensuing dielectric losses, also remain to be fully assessed (Hoult and Lauterbur, 1979; Darrasse and Ginefri, 2003). High-frequency penetration depths may also suffer from the cryogenic setup's demand to have an increased distance between the antenna and the animal. Given the larger sample losses associated with higher Larmor frequencies, it is unclear if such penalties can be mitigated by lowering the coil's thermal noise. Additional complications may result from the transceiving nature of most available cryogenic coils, for which radiofrequency field (B_1_) inhomogeneity issues may limit the number of echoing pulses that can be effectively imparted; multiple refocusing pulses at high field will also be associated with increased power depositions (SAR).

Given these considerations, it becomes clear that developing methodologies overcoming these limitations and permitting high resolution DTI, could enable a range of new applications. This study explores the performance of DTI measurements in murine brains at high fields (15.2 T) using a cryogenically cooled coil, while avoiding multiple spin echo sequences which—like RARE or GRASE—may be less effective in providing quality volumetric data when using such surface transceiver. Included in this comparison were two approaches: an optimized form of SE-EPI incorporating double sampling (Yang et al., 1996), and a SPatiotemporal ENcoding (SPEN) counterpart sequence (Ben-Eliezer and Frydman, 2011; Schmidt and Frydman, 2014; Solomon et al., 2017a). SPEN is an alternative to EPI-based acquisitions schemes, which at high fields could present a number of advantages. SPEN can be implemented in a fully ${\text{T}}_{2}^{*}$-refocused mode; in such acquisitions evolution times and refocusing pulses are timed so that intravoxel dephasings due to field inhomogeneities, are compensated throughout the course of the signal acquisition rather than at a single instant, as in conventional spin-echo experiments (Chamberlain et al., 2007; Ben-Eliezer et al., 2010; Schmidt and Frydman, 2014). Furthermore, EPI's low bandwidth along the more artifact-prone blipped dimension is defined in SPEN at the excitation stage, by a chirped pulse bandwidth that can be set at large values, thereby overcoming inhomogeneities (Ben-Eliezer and Frydman, 2011; Schmidt and Frydman, 2014; Solomon et al., 2017a). SPEN directly records its data in image space along the low-bandwidth dimension, yielding upon interleaving low-resolution but unfolded images in each scan, for which reference-less motion corrections between shots become feasible (Seginer et al., 2014; Schmidt et al., 2016; Bao et al., 2018). Last but not least, SPEN's reliance on an adiabatic swept pulse for imparting its spatial selectivity makes it robust against B_1_ inhomogeneities typical of surface coils, while allowing one to zoom without folding along the low-bandwidth dimension, thereby increasing the effective spatial resolution.

This work shows that the high-field/cryocoil combination, allows both SE-EPI and SPEN to deliver quality *in vivo* 3D DTI maps of the full mouse brain at a high (135 μm) nominal isotropic spatial resolution, in ca. 2 h—a reasonable timeframe for preclinical studies. Under the assayed conditions SNR in these full-brain experiments was generally better in SE-EPI, yet air/tissue interface artifacts were unavoidable for the latter—particularly in challenging regions like the cerebellum and the olfactory bulb (OB). By contrast, SPEN experiments delivered excellent image quality in these regions, thereby allowing a robust quantification of the diffusion tensor. Furthermore, using SPEN's ability to zoom without folding, the spatial resolution achievable in these regions could be increased, leading to DTI images with 75 μm nominal isotropic resolutions that were in full agreement with literature atlases. These capabilities were employed to investigate how morphology developed throughout the initial post-delivery weeks for the OB of mice, as evidenced by diffusivity characteristics. The whole organ evidenced maturation over weeks 1–7, as seen by both a decrease in the overall isotropic diffusivity (Apparent Diffusion Coefficient, ADC) and concurrent increase in the diffusion fractional anisotropy (FA). Within the OB, these changes were most marked for the granule cell layers at the center of the OB. These maturation changes were not evident upon inspecting the basic b0-weighted anatomical images of the organ.

## Materials and Methods

### Animal Handling

All experiments were approved by the Institutional Animal Care and Use Committee of the Weizmann Institute of Science, which is fully accredited by the AAALAC, the US NIH Office of Laboratory Animal Welfare, and the Israel Ministry of Health. For the adult rodent scans C57BL/6 female mice aged ~9 months (*n* = 3) (Envigo, Jerusalem) were used, while for the juvenile examinations C57BL/6 pups (male and female) aged 1, 2, 3, 4, and 7 week(s) were used (*n* = 2 for each age). Animals were housed in cages in a 12 h night/12 h daylight cycle, with water and food available *ad libitum;* pups stayed with their mothers until weaning. During imaging, adult mice were induced by ~3% isoflurane and then kept under ~1.3% isoflurane anesthesia mixed with 20/80% oxygen/nitrogen; their respiration was monitored throughout via a pressure sensor (SA-II, Stony Brook, NY), and maintained at 35–60 breaths per minute. A similar protocol was used for the newborns/juvenile mice, except that the initial isoflurane was in the 1–2.5% range and the anesthetic levels were set at 0.3–1%—with the concentrations adjusted according to the mice's age (from 1 to 7 weeks).

### MRI Scans

All experiments were performed on a horizontal Biospec® 15.2 Tesla Ultrashielded™ preclinical scanner equipped with an Avance IIIHD console, a B-GA 6S-100 3-axis gradient system with a 60 mm inner diameter capable of delivering a gradient strength of 1,000 mT/m, and an integrated 2nd and 3rd order shim set. DTI acquisitions on this system were performed using a surface ^1^H quadrature transmit/receive CryoProbe® surface coil with an inner diameter of 20 mm. During these acquisitions the mice heads were fixed with tooth and ears bars if aged ≥4 weeks; juvenile heads at ages 1–3 weeks were fixed with tooth but not with ears bars. All acquisitions were performed without respiratory triggering (even if respiration was always monitored). A TR of 1 second was used in all acquisitions; while shorter that the average mouse brain T_1_ at this field (≈1.8 s; Wokrina et al., 2012), this TR was chosen as compromise for fitting the full, high-resolution DTI dataset acquisition within 2 h.

**Figure 1:**
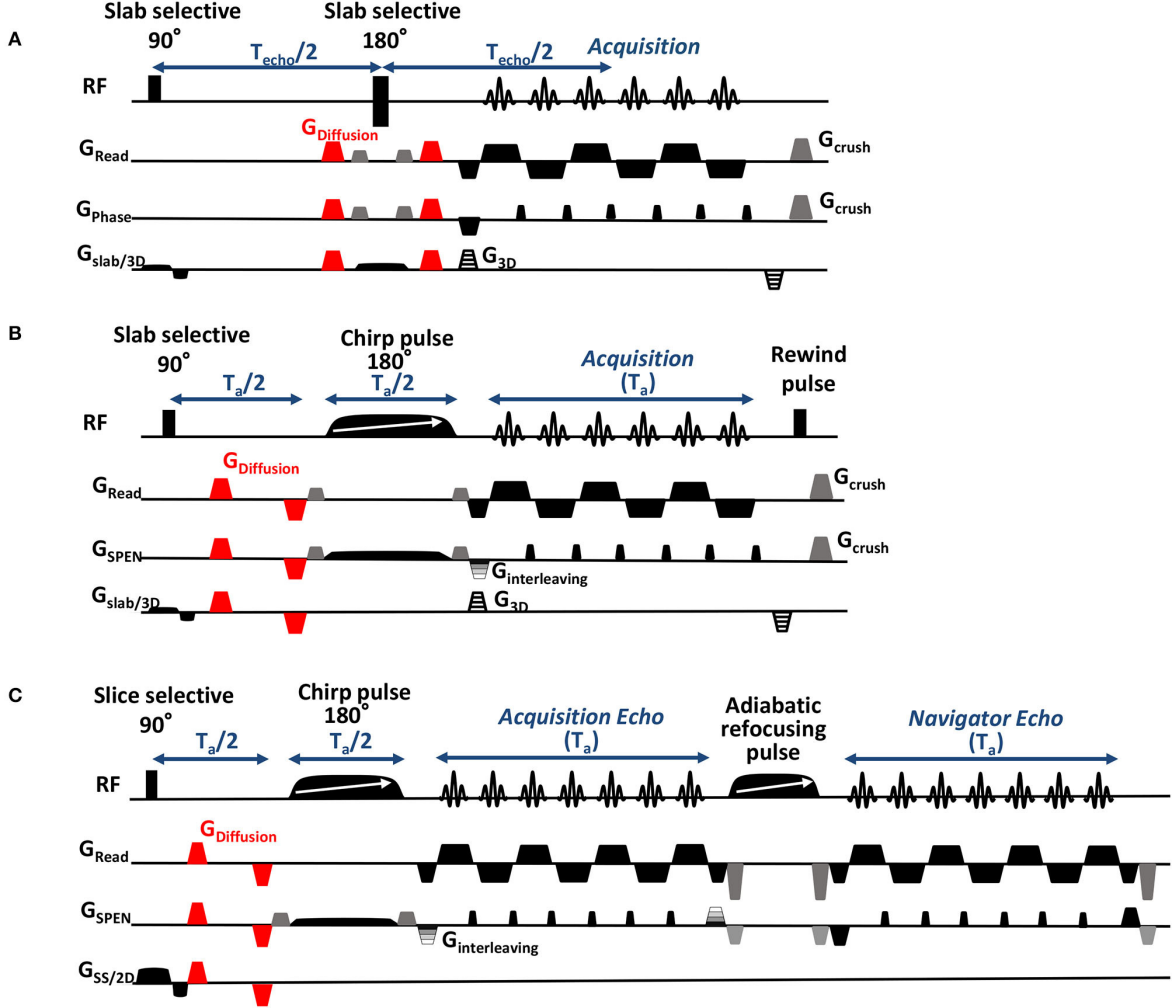
Sequences employed for: **(A)** 3D Spin Echo EPI DTI acquisitions, incorporating a phase-encoding gradient for resolving the slab dimension, a variable gradient along the low-bandwidth (phase) dimension for scan interleaving, and double sampling (double sampling involves acquiring each readout line twice in opposite directions while removing the PE blip between the oscillating gradients, in order to obtain images that are free of half-FOV ghost artifacts; Yang et al., 1996). Illustrated in red are the bipolar gradient pulses encoding the diffusion tensor. **(B)** 3D SPEN DTI acquisitions, including a 180° chirp pulse acting in the presence of a gradient that encodes the more artifact-prone, low bandwidth dimension, and a pre-encoding T_a_/2 delay introduced for achieving full-refocusing. Timings are illustrated for fulfilling SPEN's full-refocusing condition; similar interleaving and phase-encoding procedures as in the EPI counterpart were also used here. **(C)** 2D SPEN DTI sequence used to scan zoomed regions in juvenile mice, possessing similar DTI- and interleaved elements as **(B)** but including a final, 2D echo-based reacquisition of a common region in the readout/ SPEN domain for all interleaves, for correcting excessive motions in these experiments.

EPI-based DTI acquisitions were performed using the Bruker-supplied 3D sequence shown in Figure 1A, incorporating double sampling (Yang et al., 1996) to avoid characteristic half-FOV ghosting artifacts—at the expense of doubling the number of segments required vs. a SPEN-based acquisition counterpart endowed with a similar echo time. The sequence also uses scan interleaving for enhancing the resolution without compromising the FOV along the phase-encoded dimension, and a phase-encoding loop of all acquisitions for mapping the third (slab-selected) dimension. Although the possibility of relying on navigator scans to correct the half-FOV ghosts arising in the SE-EPI experiments also existed, this was found inferior to double-sampling for the relatively large number of interleaved and phase-encoded scans that were used, and therefore it was not adopted.

3D SPEN acquisitions were performed based on the sequence in Figure 1B, with encoding chirp pulses set to half of the acquisition time T_a_, in order to fulfill the full-${\text{T}}_{2}^{*}$ refocusing conditions. By contrast to EPI, phase inaccuracies between even and odd echoes were corrected in SPEN *a posteriori*, during the reconstruction stage, via a previously-described reference-less calibration method (Seginer et al., 2014; Schmidt et al., 2016; Bao et al., 2018). Also in this sequence acquisitions were interleaved along the low-bandwidth dimension, and a phase-encoding loop was used for encoding the single-slab data collected in this study (multi-slabbing not being fully supported by the scanner). Data sampling was performed throughout the full echo train acquisition, including the gradient ramps; the impact of these ramps on the spatial resolution was taken into account by suitable regridding along the readout dimension. SE-EPI data were reconstructed into 3D images on the scanner; SPEN images were reconstructed as described elsewhere (Seginer et al., 2014; Schmidt et al., 2016; Bao et al., 2018), based on a phase correction between even and odd segments and between interleaved scans, which also include motion correction and super-resolution operations. This motion correction, which is usually sufficient for dealing with adult animals and humans, was found insufficient for dealing with the multi-shot SPEN interleaving when examining the untethered newborn animals. To deal with these cases a dual-echo navigator approach (Porter and Heidemann, 2009) was added to the pulse sequence; for the sake of improving sensitivity these juvenile mice acquisitions were restricted to 2D slice-selective measurements, and employed the modified sequence shown in Figure 1C. Newborn-oriented DTI experiments, which dealt with partially mature brain characterized by higher diffusivity coefficients than mature counterparts, also relied on higher b-values to better capture the organs' morphologies. B_0_ homogeneity prior to all acquisitions was optimized using field mapping methods targeting the desired FOVs (Schneider and Glover, 1991; Vanzijl et al., 1994; Wen and Jaffer, 1995); the linewidth values obtain post-shimming as well as other basic acquisition parameters involved in these measurements are summarized in Table 1. All SPEN sequences employed together with their reconstruction pipelines are available at: https://www.weizmann.ac.il/chemphys/Frydman_group/software.

**Table 1:** Main parameters of the SPEN and EPI acquisitions and processing used in the various scans presented in this study.

**Parameters**	**Full brain SE-EPI**	**Full brain SPEN**	**Cerebellum SPEN**	**Olfactory bulb SPEN (3D)**	**Olfactory bulb development SPEN (2D)**
Repetition time (ms)	1,000	1,000	1,000	1,000	1,000
Echo Time (ms)	29.47	39.57	37.30	36.15	35.18
Acquisition time	1 h 57 min	1 h 57 min	2 h 2 min	1 h 59 min	1 h 10 min
Interleaved segments	10	5	2	2	5
Averaged scans	1	2	4	4	16
Double sampling	Yes	No	No	No	No
Field of View (mm)	15*20*5.4^$^	15*17*5.4	8*5*4	8*5*5	15*13*0.3
Matrix size	200*225*54	200*200*54	130*68*54	120*64*64	256*200*2
Regridded readout points	146	155	105	95	195
Digital resolution (μm)	103*89*100	97*85*100	76*74*74	84*78*78	77*65*300
Readout bandwidth (kHz)	500	500	250	250	500
2^nd^ dimension (Phase, SPEN) bandwidth—kHz	12.5	13.5	3.96	5.86	11.7
SPEN's time-bandwidth product	N/A	120	35	45	120
Chirp duration (ms)	N/A	8.88	8.84	7.68	10.24
Diffusion directions	12	12	16	13	23
b-values (s/mm^2^)	700	700	700	700	950
Diffusion gradient strength (mT/m)	666	666	666	666	764
Diffusion gradient duration (ms)	2	2	2	2	2
Intergradient delay (ms)	7	7	7	7	7.2
Slice selection gradient (mT/m)	8.6	8.6	9.4	11.7	156.6
FWHH (localized shimming, in Hz, ±10 Hz ^#^)	45	45	70	45	50
Denoising kernel	3*3	8*8	2*2	2*2	2*2
Average SNR of b0 image (±10%^#^)	45	21	32	25	21

$ *EPI's FOV along the blipped dimension had to be increased vis-à-vis SPEN's to avoid folding; the number of acquisition points was increased accordingly to keep equivalent spatial resolutions*.

# *Errors correspond to standard deviations through a series of measurements*.

### Post-acquisition DTI Processing

Prior to the DTI calculations all images were denoised by applying random matrix theory (Veraart et al., 2016) along all three dimensions, which improves the images without compromising resolution. The bidimensional kernels used in this denoising were optimized by acquiring and processing representative images' ROIs and evaluating the ensuing SNR; their values are summarized in Table 1. Additionally, a 3D Gaussian smoothing kernel with a standard deviation of 0.5 was applied to the denoised full brain images, blurring them by a factor of 1.35. All data were zero-filled in k-space to double the matrix size prior to Fourier transformation. The SNR of the images was computed prior to any denoising or smoothing operations, and equals the mean value of the signal divided by the standard deviation of the noise in a zone without signal (care was taken to ensure that residual EPI ghosts were not present in these regions). A relatively small b-value of ~700 s/mm^2^ was chosen for the 3D DTI adult brain studies, both to facilitate comparisons with human studies as well as for sensitivity reasons. A slightly larger b-value (~950 s/mm^2^) was used for the 2D juvenile brain DTI measurements. The diffusion tensor was then computed on Matlab R2013a with a linear combination of the log-ratio images (Basser and Pierpaoli, 1998); metrics such as the Fractional Anisotropy (FA) (Basser and Pierpaoli, 1996) and color-coded Main Diffusion Direction (MDD) (Pajevic and Pierpaoli, 1999) maps highlighting the anisotropic brain structures, were computed from the tensor.

SPEN signals along the low bandwidth dimension are inherently acquired at different echo times, and thus have different T_2_-weightings as well as different effective b-values along this axis. While the T_2_-weighting is normalized out upon computing the diffusion decay, the spatial non-uniformity in the effective b-value that derives from the different effective emission times of the spatially-resolved spin-packets over the image read out process, was taken into account in the DTI calculations (Solomon et al., 2013, 2017a).

## Results and Discussion

SNR is often the main factor that limits the spatial resolution achievable in preclinical DTI studies (Aggarwal et al., 2010; Wu et al., 2013). It is then instructive to compare the kind of sensitivity enhancements that can be observed upon porting experiments to higher fields and to cryogenically cooled coils. Supplementary Figure 1A compares representative data collected using RARE sequences at 7 T and 15.2 T, targeting a mouse's olfactory bulb region. In both cases the same room-temperature surface coil was used after suitable tuning and mechanical optimization, the same depth was targeted, and very similar acquisition parameters were employed. The increase in SNR with field is evident; the gains throughout the image are very close to the (15.2/7)^2^ ≈ 4.7 expected purely in terms of increased nuclear polarization and induction effects, with the ≈10% differences from this expectation reflecting perhaps a decreased T_2_ with field (Solomon et al., 2015). A comparison between cryocooled and room temperature coils is less straightforward, as the former includes both a cold preamplifier circuit that the latter does not have, as well as a different coil configuration. *In lieu* of this comparison, Supplementary Figure 1B presents RARE brain images obtained at 15.2 T using a 20 mm cryocoil, against counterparts obtained using a 23 mm room temperature coil and very similar acquisition parameters. The effects of a ~2.6x enhancement are again clearly noticeable, even if at the expense of a slightly more limited coverage provided by the cryocoil.

**Figure 2:**
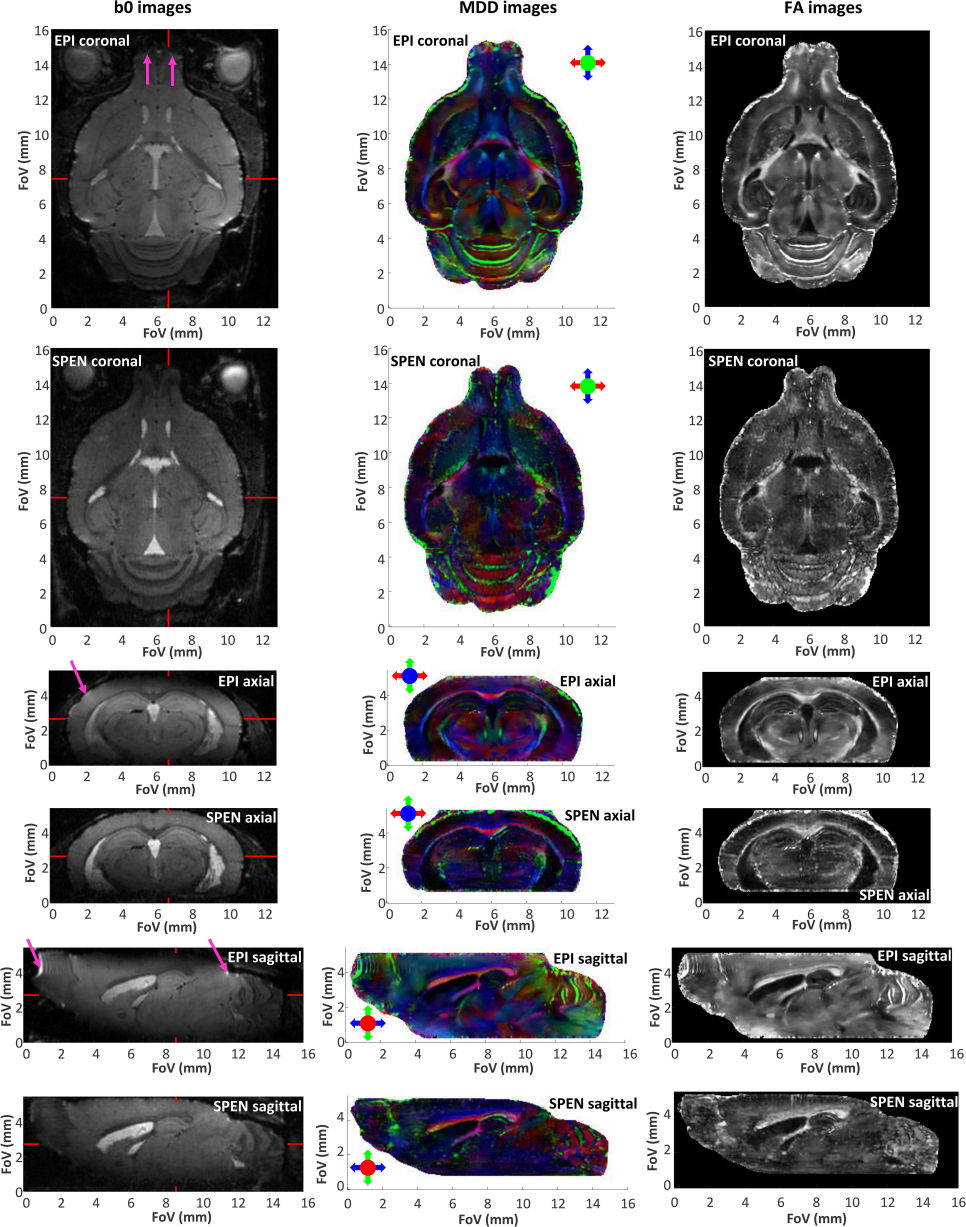
Non-weighted (b0), color-coded main diffusion directions (MDDs), and fractional anisotropy (FA) images extracted from 3D *in vivo* brain data obtained by interleaved SPEN (5 segments, 2 scans) and SE-EPI with double sampling (10 segments, 1 scan). All 3D data were collected with a cryocoil at 15.2 T with a 135 μm nominal isotropic resolution, as described in Materials and Methods and in Table 1. The read dimension corresponds to the red arrow direction, the 2nd low bandwidth (SPEN, EPI) dimension correspond to the blue arrow direction, while the phase-encoded slab encoding corresponds to the green arrow direction. Magenta arrows indicate some of the most evident distortions in the EPI images. The red markers in the b0 images indicate the positions of the orthogonal slices. See Supplementary Information for 3D video renderings of these data.

With these improvements as motivation, and while acknowledging that SNR will eventually be dependent on the experiment being assayed and the organ targeted, we set out to explore the resolution limits of DTI *in vivo* acquisitions in our 15.2 T cryocooled coil system. For animal safety reasons these acquisitions were limited to 2 h, and were performed while ensuring a sufficiently high SNR (>20) to allow us for a robust DTI reconstruction. To achieve this, data interleaving along the low-bandwidth dimension was combined with phase-encoding along the slab-selective dimension (Figures 1A,B), thus providing the desired data matrix size. Given the multi-shot nature of the ensuing experiments, head motions were mitigated using ear and tooth bars. Figure 2 presents the outcome of SE-EPI and fully-refocused SPEN DTI experiments, targeting in this manner the full brain of an adult mouse. The data are summarized as DTI results arising from coronal, sagittal and axial cuts, involving the unweighted b = 0 (b0), the color coded MDDs and the FA images –the latter two defined as per the normal DTI conventions (Basser and Pierpaoli, 1996; Pajevic and Pierpaoli, 1999). Additional videos illustrating 3D renderings of this information are presented in Supplementary Videos 1–8. Under these conditions, the SE-EPI sequence provides high quality 3D DTI data, with an average SNR of ~40 for the b0 image. This is to be compared with an SNR ≈ 21 for the SPEN counterpart. While in previous studies (Solomon et al., 2015; Cousin et al., 2019), we have been able to account for this kind of SNR differences mostly on the basis of the different effective bandwidths acting along the blipped dimension, this is not the case here: the interleaving of 10 segments provide the SE-EPI data with an encoding bandwidth of 12.5 kHz along this dimension, which is comparable with the 13.5 kHz bandwidth arising along the SPEN dimension upon using a chirped pulse bandwidth of 13.5 kHz. The lower SPEN SNR arises in this case from the relatively short T_2_s characterizing gray matter at these fields (~25 ms) (Han et al., 2019); for such values, the longer echo times demanded by SPEN's full refocusing condition (39 ms for the center of the FOV) compared to SE-EPI's TE (29 ms), explain the ensuing signal losses. Compounding these losses are the larger b ≈ 200 **s/**mm^2^ maximum weightings introduced by SPEN's imaging gradients, arising even before the application of the diffusion-encoding bipolar modules (Solomon et al., 2013). These biases in b-weightings and TEs might account for the slight differences noticeable in Supplementary Figure 2, which compares average ADC and FA distributions afforded by the two methods on the same animal.

These considerations notwithstanding, it is worth remarking the distorting effects that are imparted by field inhomogeneities on some regions of the SE-EPI data; including in the olfactory bulb, the cerebellum as well as close to the cortical surface, as is clearly visible in the sagittal slices. Supplementary Figure 3 displays some of these features with a comparison between two sagittal profiles extracted from SE-EPI and SPEN images, at selected positions. Field-inhomogeneity distortions are evidenced in these normalized 1D profiles, as are more subtle susceptibility-derived effects as well. In the cerebellum, for instance, the field inhomogeneities induce a degree of overlap of the WM and GM signatures, shifting the former signals in the SE-EPI data. This leads to minor artifacts in the ensuing MDD images, where some regions transitions from green to red—where they should be uniformly red (Baloch et al., 2009; Aggarwal et al., 2010), as they appear in the SPEN-derived data. Notice that these artifacts arise despite the use of shimming up to 3rd order, and of a high encoding bandwidth resulting from the use of ten interleaved segments. The fact that these distortions are less apparent in the corresponding SPEN images is not a result of a larger bandwidth along the vulnerable dimension, but rather reflecting the extra robustness to ${\text{T}}_{2}^{*}$-derived decays and pile-ups that arise upon using the full refocusing conditions.

**Figure 3:**
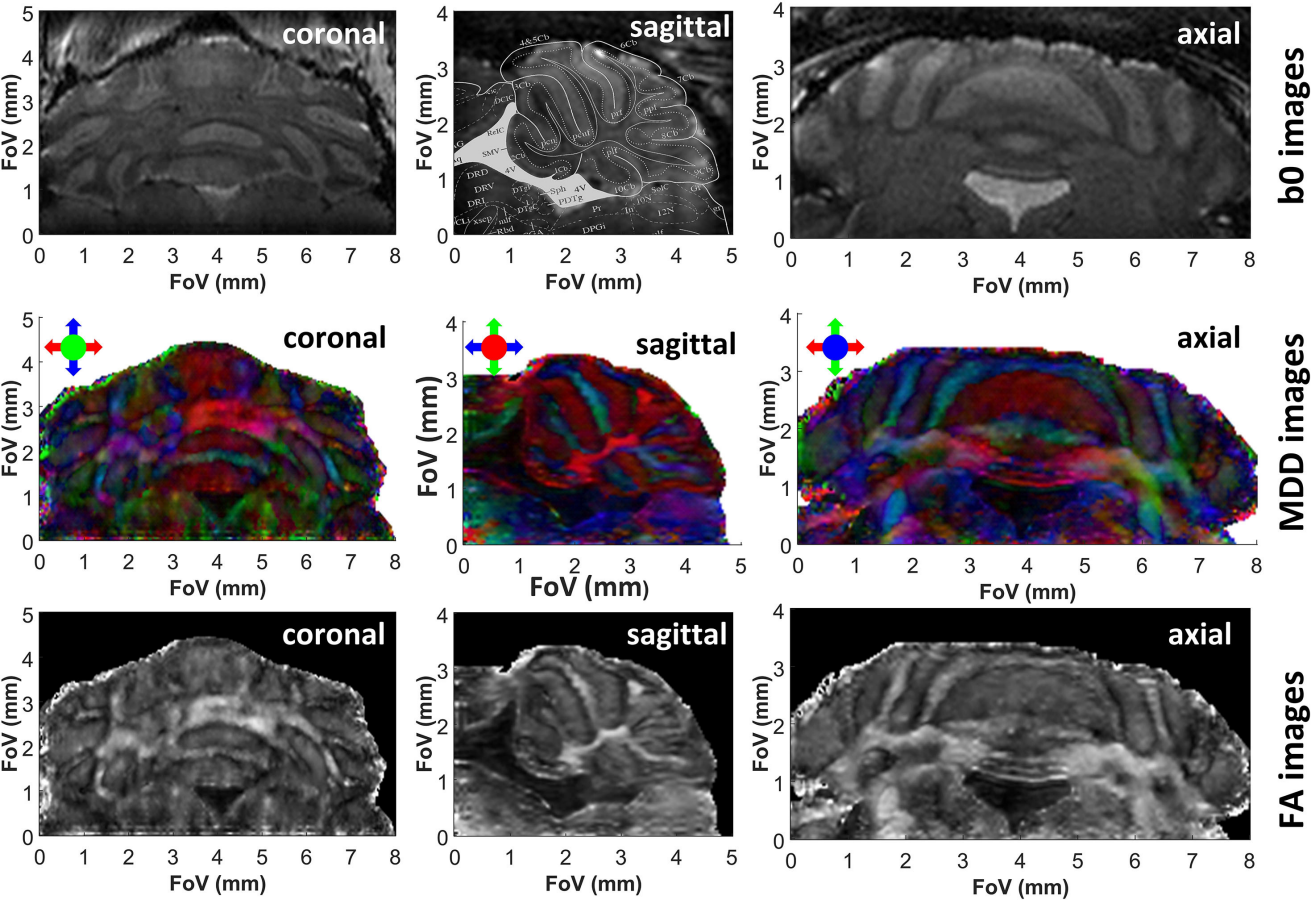
b0, color-coded MDD and FA images extracted from 3D SPEN *in vivo* brain experiments zooming in the cerebellum, at a 75 μm nominal isotropic resolution. Each image is identified by the orientation of the shown slice. The read dimension corresponds to the red arrow direction, the 2nd phase and low bandwidth dimension correspond to the blue arrow direction and the slab with constant time phase encoding correspond to the green arrow direction. Superimposed on the sagittal b0 image are contours extracted from the Paxinos and Franklin mouse brain atlas (Franklin and Paxinos, 2008) at a lateral position of 0.24 mm, evidencing the close correspondence between the features revealed by the DTI *in vivo* measurements and those evidenced by *ex vivo* histology. See Supplementary Information for 3D renderings of these data.

**Figure 4:**
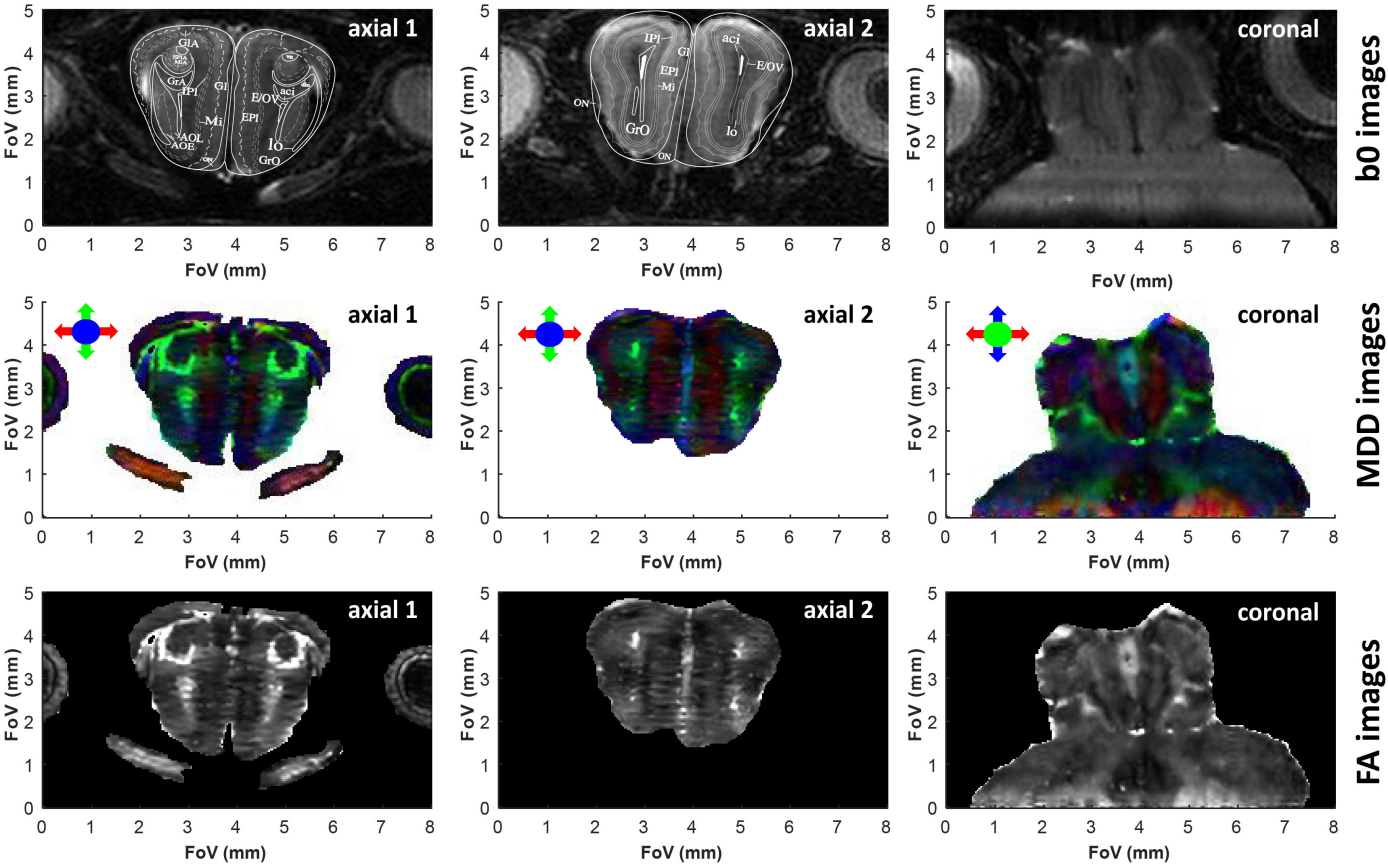
Similar as Figure 3, but upon zooming onto the olfactory bulb. In lieu of less informative sagittal images, we show two axial slices taken at different heights within the olfactory bulb. In this acquisition the read dimension corresponds to the red arrow direction, the 2^nd^ (low bandwidth) SPEN dimension corresponds to the blue arrow direction, and the 3^rd^ phase encoded dimension corresponds to the green arrow direction. SPEN's zoom-in ability is evidenced in the coronal slice, where it allows focusing on the olfactory bulb area without folding from the rest of the brain. Superimposed to these two axial slices are the images extracted from the Paxinos and Franklin mouse brain atlas (Franklin and Paxinos, 2008) at Bregma position of 3.56 and 4.28 mm for slices 1 and 2, respectively. The subtle horizontal stripes present in the axial FA images originate from minor phase disturbances between odd and even echoes and/or segments, limiting the self-referenced post-processing phase correction in the SPEN reconstruction. See Supplementary Information for 3D renderings of these data, and the main text for the meaning of the various layers in the atlas.

As demonstrated in previous studies (Wheeler-Kingshott et al., 2002; Finsterbusch, 2009; Wu et al., 2014; Samson et al., 2016), DTI's resolution may be improved by zooming on specific regions of the brain, without excessive degradations in the SNR achievable per unit time. Zooming multiple dimensions of a DTI experiment within an object, however, is compromised by folding complications along the phase-encoded dimensions: doing so while using k-encoded sequences like EPI requires performing special manipulations that are non-trivial and susceptible to inhomogeneity artifacts –for instance excitations with selective multidimensional pulses (Wu et al., 2014), or zonally magnified acquisitions (Mansfield et al., 1988). One of SPEN's advantages rests in its ability to zoom on specific regions of the brain without folding (Li et al., 2015), simply by confining the bandwidth of the chirp pulse encoding the spatial positions. This in turn, brings ensuing improvements in sensitivity. Figures 3, 4 demonstrate the potential of this approach to increase DTI's spatial resolution while preserving robustness vs. magnetic field inhomogeneities and achieving sufficient sensitivity, with applications to studies on the cerebellum and the olfactory bulb, respectively. These images, which show no appreciable folding artifacts along any dimension, were collected with an optimized time-bandwidth product for the chirp encoding pulse that avoided artifacts despite the inhomogeneities arising in these brain regions at high fields. At the same time, time-bandwidths were chosen small enough to give acceptable SNR values of 32 and 25 in the b0 images, while achieving nominal spatial resolutions of 75 and 80 μm isotropic for the two organs. Also shown superimposed on the b0 images are uniformly scaled maps taken from the Paxinos and Franklin *ex vivo* murine brain atlas (Franklin and Paxinos, 2008), illustrating various anatomical divisions for these regions. For the cerebellum (Figure 3) this sagittal overlay highlights the characteristic foliation pattern of the organ, with the multiple foldings of the ten lobules of the cerebellum clearly discernible. The central vermis flanked by two symmetrically, highly patterned hemispheres are also clearly visible in the axial and coronal cross-sections. A high level of congruence between the *in vivo* DTI data and *ex vivo* Atlas structures is also evidenced by the main olfactory bulb's MDD images (Figure 4, center panels). While not showing as strong a directionality as the cerebellum—as expected from the dominance of gray matter in the olfactory bulb—the images show multiple fine details. The glomerular, mitral and external plexiform layers evidence low anisotropy, which is compatible with their large fraction of cell bodies. Glomeruli are also visible and are also characterized by low anisotropy, enveloped by structures such as the anterior commissure and the lateral olfactory tract—each layer with a slightly distinct anisotropy.

**Figure 5:**
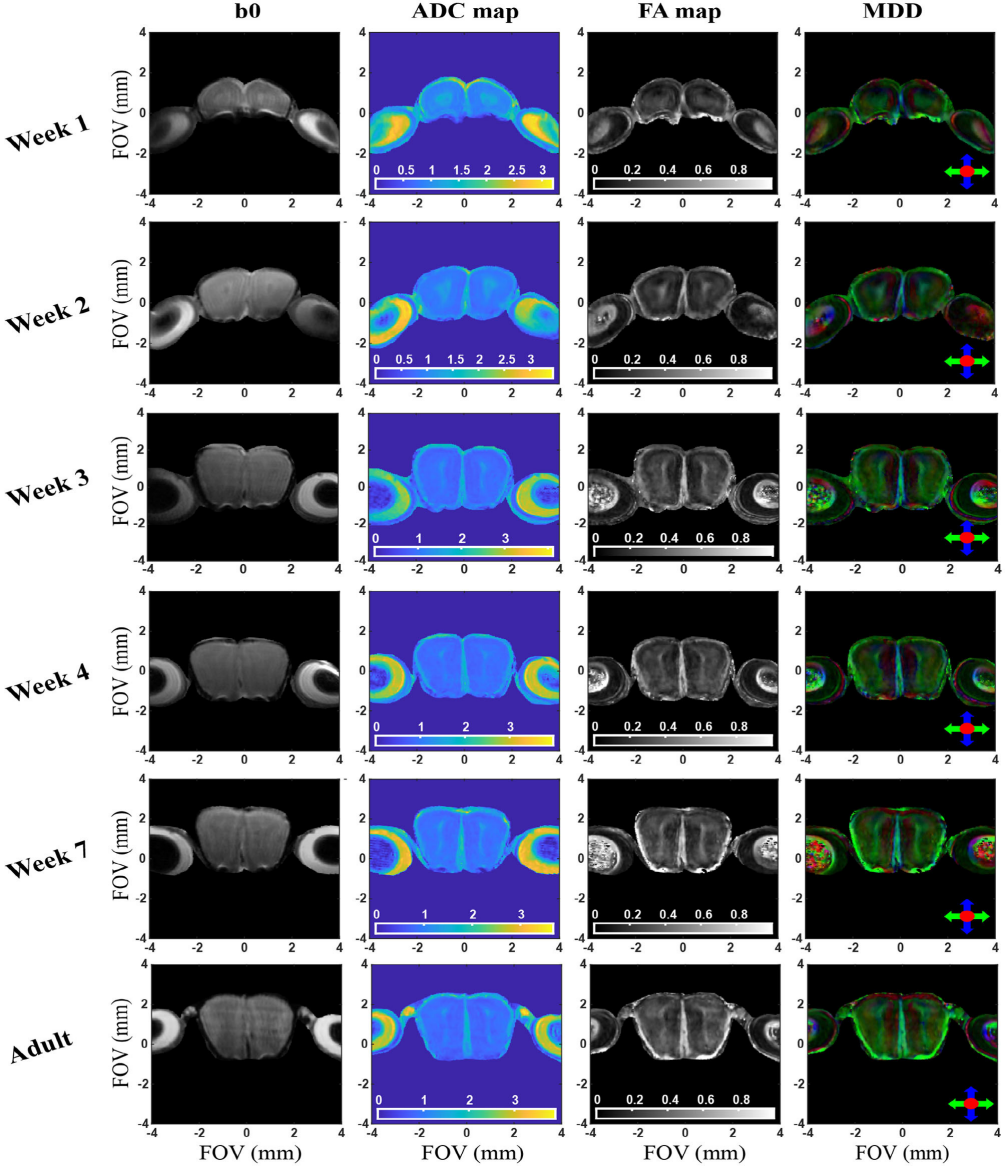
b0, ADC, FA, and MDD images extracted from *in vivo* 2D SPEN brain experiments for a representative slice/animal, focusing on the OB region. Axes indicate dimensions in mm; ADCs are mapped in units of 10^−3^ mm/s^2^. See text for additional experimental details and Supplementary Information for additional slices / animals. The curvature observed in the bottom of the OB/eyes axis appearing to decrease with age, arises from field inhomogeneities.

In view of the ability of these experiments to tackle the OB's microstructure, we decided to investigate the development of this organ with age. This in turn complements past MRI studies that have focused on mouse brain development by diffusion and perfusion (Mori et al., 2001; Zhang et al., 2003; Wadghiri et al., 2004; Chuang et al., 2011). *In vivo* DTI was longitudinally performed from the first post-delivery week till adulthood; as illustrated in Supplementary Figure 4, quality b-weighted images could then be obtained by SPEN but not by SE-EPI, even for the largest juvenile animals (which were substantially more amenable to study than younger neonatal cases). Figure 5 and Supplementary Figure 5 summarize observations from these measurements. Given the challenges involved in scanning newborn mice, the high isotropic resolution in Figures 2–4 was not pursued by these studies. These focused instead in multi-slice analyses based on the sequence in Figure 1C: increasing each slice's thickness to 300 μm allowed us to retain a sufficiently high in-plane resolution (77 × 65 μm), to enable the discernment of changes within these small structures. These slices were chosen along a coronal orientation in order to facilitate comparisons with adult 3D data as well as with Atlas data (Franklin and Paxinos, 2008); as further described in Methods, not being able to fix the pups' heads with ear bars also called for additional provisions in terms of navigator-aided motion compensation.

**Figure 6:**
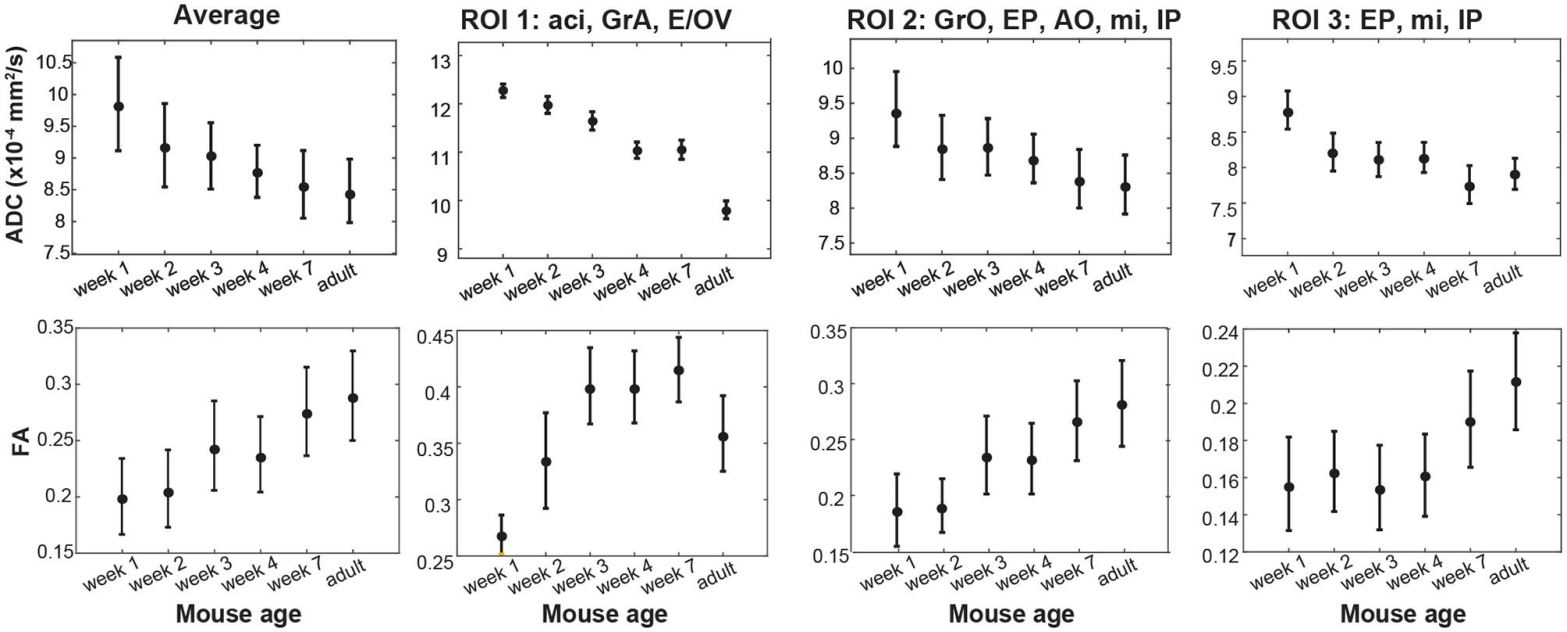
Isotropic ADC and FA maturation for the OB and for labeled ROIs as illustrated in Supplementary Figure 6, observed for different animals as a function of age. Two different animals were scanned for each age; 12 different animals were thus scanned in the study. Bars represent the scattering measured for each observable within each OB/ROI for each age, rather than errors the measurements. See the main text for constituents and abbreviations used in these different ROIs.

Besides an obvious increase in the OB's size, the DWI data in Figure 5 and Supplementary Figure 5 evidence systematic area-specific changes with age, that are absent in the b0 images but are reflected from the ADC, FA and MDD maps. To better quantify these changes, the structure of the OB suggests to segment them based on anatomically-defined layered structures. Three Regions-of-Interest (ROI) were thus defined for each lobe and for every slice in these OB analyses, based on the MDD and Atlas analyses indicated in Figure 4 and Supplementary Figure 6. The first of these ROIs (#1) corresponded to the center region of the lobes, including the anterior commissure (aci), the Granule cell layer of the accessory olfactory bulb (GrA), and the ependymal and subependymal layer and the olfactory ventricle (E/OV). The second ROI (#2) corresponds mainly to the OB's glomerular layer and partly to external granular cell (GrO) and external plexiform (EP) layers, the mitral cells (mi) and the anterior olfactory nucleus (AO). The third, innermost ROI (#3) appears as the least anisotropic part of the lobe, and should correspond mainly to GrO, mi and internal plexiform (IP) layer. Figure 6 shows the evolution of the ADC and FA arising from these ROIs, as well as the scattering of these observables within the selected regions for the animals studied. Each dot represents an average, with two animals measured for each age. Error bars describe the standard deviation of the diffusion metrics arising from these measurements within each ROI, and are thus a reflection of heterogeneity in each region of the organ, rather than actual errors in the measurements. Also shown is the evolution of these parameters for the OBs taken in unison. The maturation of this organ seems to be associated with an overall decrease in ADC and increase in FA values –likely explained by a systematic growth in the number and the complexity of OB's circuitry (Pomeroy et al., 1990). A similar pattern has been observed for the evolution of mice brains as a whole (Verma et al., 2005; Larvaron et al., 2007; Bao et al., 2020). These changes, however, are not uniform, and seem to be most significant for the GrA region at the center of the lobes. Notice how ROI1 elongates and becomes thinner with age, while exhibiting the slowest diffusivity and largest anisotropy among the various regions. This region also evidences the largest changes throughout the maturation process, including an increase in the number of cell bodies, a prolongation of dendrites' length and complexity, and overall size (Jacque et al., 1985; Pomeroy et al., 1990). In addition, the DTI metrics of all ROIs are likely reflecting the extensive myelination of the mouse brain, that only onsets around 8 days of age: the decrease in ADC thus results from a combination of increased myelin and increased dendritic complexity. While several previous studies have verified such features *ex vivo*, the results provided here reveal the potential of performing such diffusivity-based mapping on OBs *in vivo* and on the same animal cohort.

## Conclusions

The present study explored a number of alternatives that may help the implementation of high-resolution DTI studies under *in vivo* preclinical conditions. Common to these alternatives were the use of high fields and of cryogenically cooled coils –both of which delivered substantial sensitivity enhancements under realistic *in vivo* conditions, when compared against lower field and conventional thermal counterparts. As counterparts to these measuring tools two relatively simple sequences that are not highly sensitive to the RF inhomogeneities associated to the use of cryo-cooled surface coil transceivers, were employed in the DTI measurements: 3D SE-EPI with double sampling, scan interleaving and phase encoding; and fully-refocused 3D SPEN with scan interleaving and phase encoding. Despite initial concerns for the susceptibility distortions that might arise at ultrahigh fields and the challenges associated with data interleaving, SE-EPI performed remarkably well: its use of double sampling permitted the integration of the multiple DTI scans without any noticeable ghosting, and the presence of third-order shims provided sufficient global field homogeneity to capture the majority of the brain without distortions. Thus, when comparing full brain DTI acquisitions, SE-EPI provided a superior SNR over SPEN counterparts –affected due to their nature by longer echo times for identical FoVs and spatial resolutions, and by higher b-values associated to the demands of the imaging gradients themselves. Still, this acquisition scheme proved more resilient to magnetic field inhomogeneity, particularly in regions bordering tissue/air interfaces. A particularly interesting option arose when coupling this ability with SPEN's capacity to zoom without folding; this allowed us to increase the spatial resolution without raising acquisition times, and focus on specific brain regions such as the cerebellum and olfactory bulb. Agreement with Atlas data was then excellent. It remains to be seen how these DTI experiments compare to alternatives that rely more heavily on multiple RF pulses, including RARE/FSE and GRASE sequences, when attempting to perform high-resolution DTI experiments; it also remains to be seen whether the performance of SE-EPI might still be improved by the use of advanced correction methods.

Using SPEN-based DTI as new tool, attention could also be focused on the development of mice's olfactory bulbs. Quality, consistent data could be obtained from this organ, which we analyzed in terms of the evolution of sub-mm layers from age 1 week onwards. According to the DTI parameters, this showed a differential maturation that affected most markedly internal layers associated to the granule cells of the accessory OB. Efforts to translate these morphological changes into functional ones are also underway. The SPEN-based DTI acquisition sequences here produced could also serve as “templates” for additional biologically-relevant experiments, including measurements of cell body density (Palombo et al., 2020), axon diameter (Veraart et al., 2020), kurtosis, or other measures of local microstructure (Yon et al., 2020). Such tests, as well as comparisons with other approaches such as xSPEN (Solomon et al., 2017b; Zhang et al., 2018), are in progress. Also under investigation is how can these sequences target challenging brain regions while dealing with B_1_ inhomogeneities, when performing high-resolution DTI studies on humans at ultrahigh fields like 7T. These tests will be complicated by the higher SAR of chirped pulses over conventional counterparts, a complication that increases linearly with the targeted sweep bandwidth. Solutions to this problem are also being sought.

## Data Availability Statement

All datasets generated for this study are included in the article/Supplementary Material.

## Ethics Statement

All experiments were approved by the Institutional Animal Care and Use Committee of the Weizmann Institute of Science, which is fully accredited by the AAALAC, the US NIH Office of Laboratory Animal Welfare, and the Israel Ministry of Health.

## Author Contributions

MY, NS, and LF conceived the study. MY, QB, and OC performed experiments. MY, QB, OC, and RH provided methods for analysis and analyzed data. MY, NS, and LF evaluated the results and wrote manuscript.

## Conflict of Interest

The authors declare that the research was conducted in the absence of any commercial or financial relationships that could be construed as a potential conflict of interest.

## Abbreviations

ADC: Apparent diffusion coefficient
EPI: Echo Planar Imaging
DWI: Diffusion weighted imaging
DTI: Diffusion tensor imaging
FA: Fractional anisotropy
FOV: Field of View
FSE: Fast spin echo
FT: Fourier transformation
MDD: main diffusion directions
PE: Phase encoding
OB: Olfactory bulb
ROI: Region of Interest
SE: Spin echo
SNR: Signal-to-noise ratio
SPEN: Spatiotemporal encoding.
