# Supplementary material for: High-Resolution 3D in vivo Brain Diffusion Tensor Imaging at Ultrahigh Fields: Following Maturation on Juvenile and Adult Mice
Source: Front Neurosci. 2020 Nov 20;14:590900. doi: 10.3389/fnins.2020.590900 (PMC7714913; doi:10.3389/fnins.2020.590900)
Supplement: Supplementary file 1 [file Data_Sheet_1.DOCX]

**Supplementary information for**

**High-resolution 3D *in vivo* brain diffusion tensor imaging at ultrahigh fields: Following maturation on juvenile and adult mice**

**Maxime Yon^1^, Qingjia Bao^1^, Odélia Jacqueline Chitrit^1^, Rafael Neto Henriques^2^, Noam Shemesh^2^, and Lucio Frydman^1^**

*^1^Department of Chemical and Biological Physics, Weizmann Institute, Rehovot, Israel*

*^2^Champalimaud Research, Champalimaud Centre for the Unknown, Lisbon, Portugal*


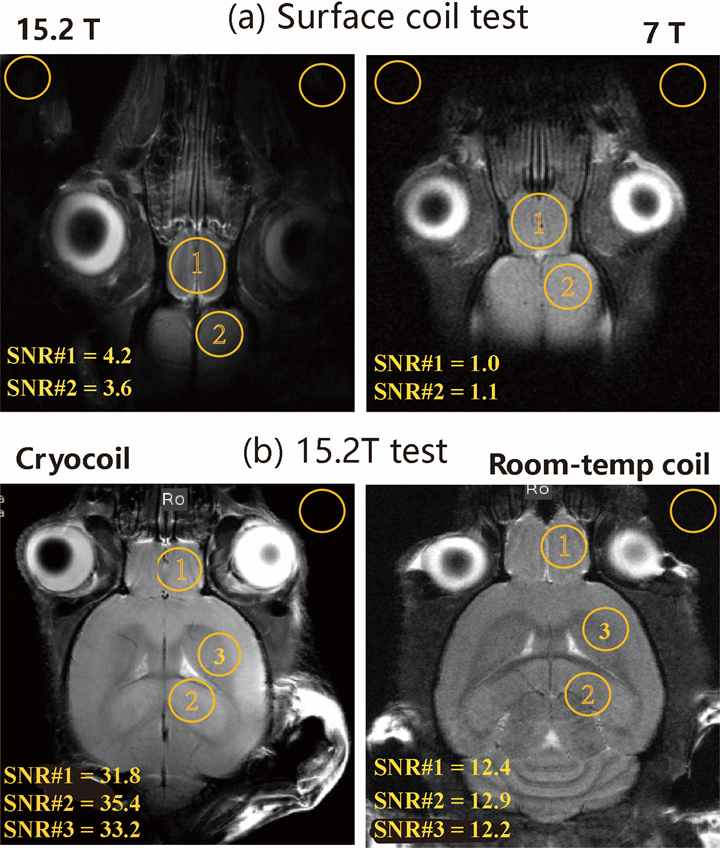


**Figure S1:** Effects of field (a) and of cryogenic coil/preamp cooling (b) on SNR, as measured in vivo on a mouse’s head. All data were recorded using RARE sequences with RARE_Factor = 8, 2 averages, TR/TE = 2000/32 ms; spatial resolutions were 0.11x0.11x0.6 mm^3^ for surface-coil experiments (a), and 0.04x0.04x0.5 mm^3^ for cryo-coil experiments (b). The 7 T image was recorded on a DD2^®^ 7T/110mm horizontal magnet scanner (Agilent Technologies, Santa Clara, CA) equipped with a 1000 mT/m maximum gradient triple-axis setup. The set in (a) was recorded using the same 15 mm coil centered on the animal’s olfactory bulb –suitably tuned, at the corresponding Larmor frequencies of 299.9 and 650.1 MHz respectively. As similar sensitivity profiles were observed at both frequencies, the same regions could be sampled at the two fields. The set in (b) used a 20 mm cryocoil (left) and 23 mm room temperature coil (right). Signal and noise were recorded for the different parts of the images indicated by the yellow circles; for these regions SNR/nL were 3.9±0.3 and 1.0±0.1 at 15.2 and 7T respectively (a); 34.5±1 and 12.5±0.**3** for the cryo- and room-temperature coils, respectively (b).


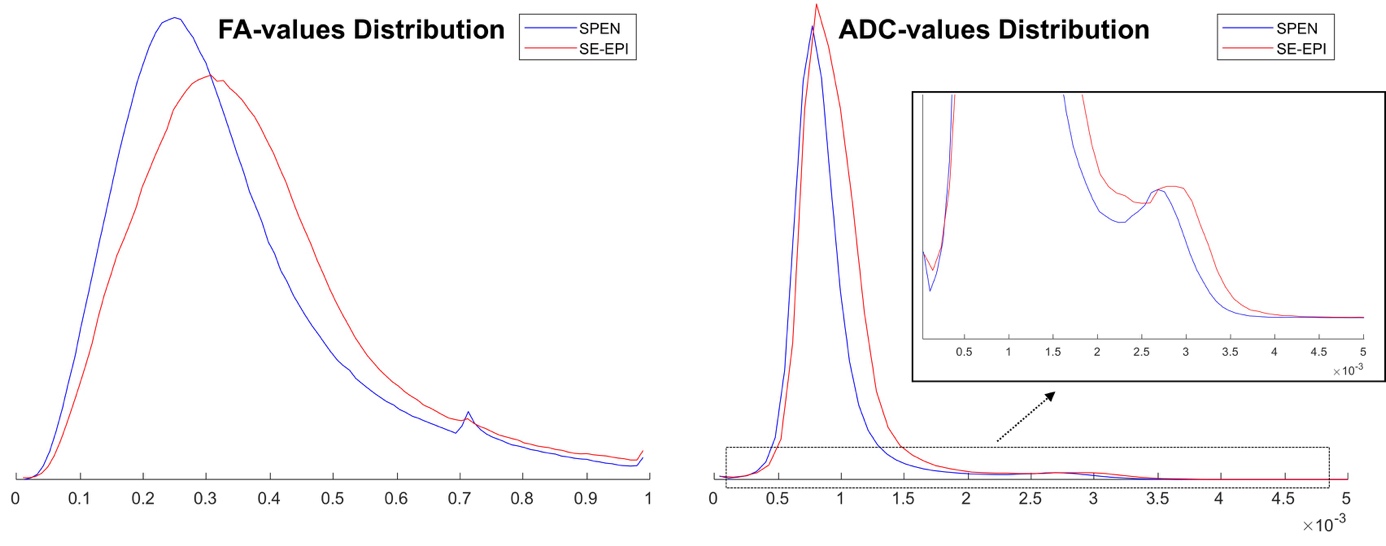


**Figure S2:** Comparisons between distributions afforded by SPEN and SE-EPI for the FA and ADC values for the whole-brain results summarized in Figure 2, main text.


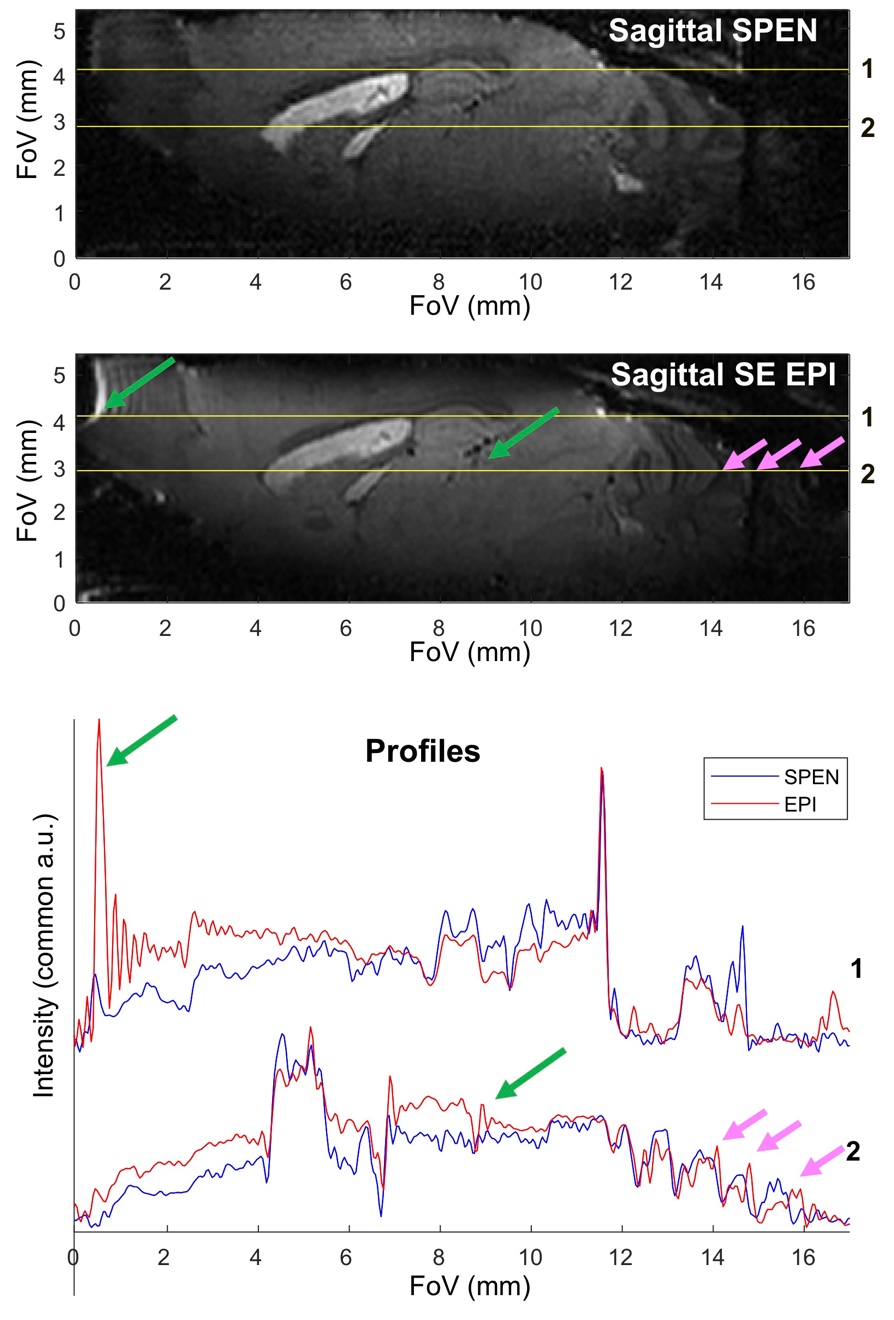


**Figure S3:** Comparisons between SPEN (top) and SE-EPI (center) b0 sagittal slices. The bottom panel compares 1D profiles extracted from the SPEN and SE-EPI b0 images on top (blue and red, respectively) at the yellow lines marked 1 and 2, as indicated. Green arrows point to challenging B_0_ inhomogeneity regions; magenta arrows point to SE-EPI miss-registrations.

**Figure S4:** b0 and b-weighted images arising from 2D SPEN and SE-EPI experiments focusing on the OB region of a 7-week-old mouse. SPEN acquisition conditions are as detailed in Materials and Table 1; EPI relied on similar parameters: slice thickness and resolutions (60x65x300µm^3^), 4 segments, 10 averages, 10 and 500 kHz phase and readout bandwidths, respectively.

**Figure S5:** b_0_, ADC, FA, and MDD images extracted from *in vivo* 2D SPEN brain experiments for the OB region at different development stages. Data complements the information shown in Figure 5 (main text), and similar considerations apply.

**Figure S6:** b_0_ images extracted from 2D SPEN olfactory bulb experiments on an animal as a function of its development, showing the color-coded ROI segmentation used in the main text to obtain the data in Figure 6. Shown on top are the main constituents of each ROI: anterior commissure (aci), Granule cell layer of the accessory olfactory bulb (GrA), ependymal and subependymal layer and the olfactory ventricle (E/OV), external granular cell (GrO), external plexiform layer (EP), mitral cells (mi), anterior olfactory nucleus (AO), internal plexiform layer (IP).

**Video 1:** Axial slider of the non-b-weighted full mouse brain 3D image acquired with a Spin Echo (SE) double-sampled EPI sequence (main paper, Fig 1A). The image has a spatial resolution of 139*120*135 µm along the read, phase and slab directions respectively. The read direction corresponds to the antero-posterior axis, the phase direction to the left to right axis, and the slab direction to the superior-inferior axis.

**Video 2:** Axial slider of the color-coded main diffusion directions (MDDs) of the full mouse brain 3D image acquired using a spin-echoed double-sampled EPI acquisition (Fig. 1A). The image has a spatial resolution of 139*120*135 µm along the read, phase and slab directions respectively. The read direction corresponds to the antero-posterior axis and it is color coded in blue, the phase direction corresponds to the left-to-right axis and is color coded in red, and the slab direction to the superior-inferior axis and is color coded in green.

**Video 3:** Axial slider of the non-b-weighted full mouse brain 3D image acquired with a fully T_2_*-refocused SPEN acquisition (main text, Fig. 1B). The image has a spatial resolution of 131*115*135 µm along the read, phase and slab directions respectively. The read direction corresponds to the antero-posterior axis, the phase direction to the left-to-right axis, and the slab direction to the superior-inferior axis.

**Video 4:** Axial slider of the color-coded main diffusion direction (MDD) for a full mouse brain 3D image acquired with a fully T_2_*-refocused SPEN acquisition (Fig. 1B). The image has a spatial resolution of 131*115*135 µm along the read, SPEN and slab directions respectively. The read direction corresponds to the antero-posterior axis and is color coded in blue, the phase direction to the left-to-right axis and is color coded in red, and the slab direction to the superior-inferior axis is color coded green.

**Video 5:** Sagittal slider of the non b-weighted 3D image zoomed on the cerebellum acquired with a fully T_2_*-refocused SPEN acquisition. The image has a spatial resolution of 76*74*74 µm along the read, SPEN and slab directions respectively. The read direction corresponds to the left-to-right axis, the phase direction to the antero-posterior axis, and the slab direction to the superior inferior axis.

**Video 6:** Sagittal slider of the color-coded main diffusion direction 3D image zoomed on the cerebellum acquired with a fully T_2_* refocused SPEN acquisition. The image has a spatial resolution of 76*74*74 µm along the read, SPEN and slab directions respectively. The read direction corresponds to the left-to-right axis and is color coded in red, the SPEN direction to the antero-posterior axis and it is color coded in blue, and the slab direction to the superior inferior axis it is color coded in green.

**Video 7:** Axial slider of the non b-weighted 3D image zoomed on the olfactory bulb acquired with a fully T_2_* refocused SPEN acquisition. The image has a spatial resolution of 84*78*78 µm along the read, SPEN and slab directions respectively. The read direction corresponds to the left-to-right axis, the phase direction to the antero-posterior axis, and the slab direction to the superior-inferior axis.

**Video 8:** Axial slider of the color-coded main diffusion direction 3D image zoomed on the olfactory bulb acquired with a fully T_2_* refocused SPEN acquisition. The image has a spatial resolution of 84*78*78 µm along the read, SPEN and slab directions respectively. The read direction corresponds to the left-to-right axis and it is color coded in red, the SPEN direction to the antero-posterior axis and is color coded in blue, and the slab direction to the superior-inferior axis and is color coded in green.
